# Development, confirmation, and application of a seeded *Escherichia coli* process control organism to validate *Salmonella enterica* serovar Typhi environmental surveillance methods

**DOI:** 10.1371/journal.pone.0301624

**Published:** 2024-05-07

**Authors:** Sarah E. Philo, Nicolette A. Zhou, Lorraine M. Lillis, Venkata Raghava, Dilip Abraham, Vinoth Kumar, Nirmal Kumar, Jonathan Rigby, Joanna Ciol Harrison, Christine S. Fagnant-Sperati, Alexandra L. Kossik, Angelo Q. W. Ong, Rachael Swanstrom, Elisabeth Burnor, Bethel Demeke, Nicola K. Beck, Jeffry H. Shirai, Stephen J. Libby, David S. Boyle, Nicholas Feasey, Gagandeep Kang, John Scott Meschke

**Affiliations:** 1 Department of Environmental and Occupational Health Sciences, University of Washington, Seattle, Washington, United States of America; 2 PATH, Seattle, Washington, United States of America; 3 Christian Medical College Vellore, Vellore, India; 4 Malawi Liverpool Wellcome Programme, Kamuzu University of Health Sciences, Blantyre, Malawi; 5 Department of Laboratory Medicine and Pathology, University of Washington, Seattle, Washington, United States of America; 6 Department of Clinical Sciences, Liverpool School of Tropical Medicine, Liverpool, United Kingdom; Aga Khan University, PAKISTAN

## Abstract

*Salmonella enterica* serovar Typhi (*S*. Typhi) is the causative agent of Typhoid fever. Blood culture is the gold standard for clinical diagnosis, but this is often difficult to employ in resource limited settings. Environmental surveillance of waste-impacted waters is a promising supplement to clinical surveillance, however validating methods is challenging in regions where *S*. Typhi concentrations are low. To evaluate existing *S*. Typhi environmental surveillance methods, a novel process control organism (PCO) was created as a biosafe surrogate. Using a previous described qPCR assay, a modified PCR amplicon for the *staG* gene was cloned into *E*. *coli*. We developed a target region that was recognized by the Typhoid primers in addition to a non-coding internal probe sequence. A multiplex qPCR reaction was developed that differentiates between the typhoid and control targets, with no cross-reactivity or inhibition of the two probes. The PCO was shown to mimic *S*. Typhi in lab-based experiments with concentration methods using primary wastewater: filter cartridge, recirculating Moore swabs, membrane filtration, and differential centrifugation. Across all methods, the PCO seeded at 10 CFU/mL and 100 CFU/mL was detected in 100% of replicates. The PCO is detected at similar quantification cycle (Cq) values across all methods at 10 CFU/mL (Average = 32.4, STDEV = 1.62). The PCO was also seeded into wastewater at collection sites in Vellore (India) and Blantyre (Malawi) where *S*. Typhi is endemic. All methods tested in both countries were positive for the seeded PCO. The PCO is an effective way to validate performance of environmental surveillance methods targeting *S*. Typhi in surface water.

## Introduction

*Salmonella enterica* serovar Typhi, *S*. Typhi, is a gram-negative bacterium that causes Typhoid fever [1]. *S*. Typhi are transmitted via the fecal-oral route, and most individuals become infected after ingesting contaminated food or water [2]. Symptoms of infection include fever, headache, malaise, and gastrointestinal symptoms [1,3]. Despite rising antibacterial resistance, *S*. Typhi infection is treatable with antibiotics and preventable with two different vaccines [1]. Risk of infection in high-income countries is low compared to the risk of infection in low- and middle-income countries (LMICs), particularly in children under five living in poverty [4,5].

Traditionally, surveillance for *S*. Typhi is carried out at the clinical level when infected individuals come into contact with the healthcare system [1,6]. Cases are diagnosed after isolating the bacteria via blood culture [1,3]. However, clinical surveillance drastically underestimates the true burden of *S*. Typhi and other diarrheal diseases because a large proportion of infected individuals do not access the healthcare system or blood culture testing is unavailable [7]. Additionally, the widespread availability of antibiotics in LMICs reduces the sensitivity of blood culture diagnostics as patients often consume antibiotics prior to blood culture [1]. Lastly, because blood culture capacity remains in short supply in Typhoid endemic regions, environmental surveillance (ES) is a promising approach to the surveillance of *S*. Typhi [8]. ES has been a crucial tool in the Global Polio Eradication Initiative (GPEI), as it has been shown to detect poliovirus weeks before individuals with acute flaccid paralysis are diagnosed as clinical cases [9–11]. The GPEI was founded in 1988 after a declaration by the World Health Assembly to globally eradicate polio by the year 2000 [12]. While that goal was not achieved, substantial progress has been made. Wild poliovirus (WPV) serotypes 2 and 3 have been eradicated, serotype 1 transmission continues in only two countries: Afghanistan and Pakistan, and WPV cases have dropped by more than 99.9% [13]. ES has also been used around the world in response to the COVID-19 pandemic. Early in the pandemic, it was suggested that ES could help indicate when SARS-CoV-2 entered a new community or to detect changes in infection trends [14]. More recently, ES is being used to track SARS-CoV-2 variants [15–17].

Microbiological controls to determine if ES methods are functioning as required would be greatly beneficial when applying ES for *S*. Typhi detection. A similar control has been developed for poliovirus ES methods, with a 30% sample positivity rate at each site for the isolation of non-polio enteroviruses [10,18]. To better understand performance of *S*. Typhi ES methods, we sought to develop and validate an internal process control organism (PCO) in a biosafe K-12 strain of *E*. *coli* using a modified qPCR target gene for *S*. Typhi to utilize as a seeded matrix spike, or a target or organism added into a sample that is not endogenous to the sample to serve as a quantifiable control. A commonly used *S*. Typhi qPCR protocol was multiplexed to detect both the original and modified targets. The PCO and *S*. Typhi were next seeded into lab-based *S*. Typhi ES methods to evaluate the methods’ efficacy at concentrating and detecting these bacteria. The PCO was detectable at levels equal to or lower than what would be expected in environmental samples containing *S*. Typhi. Additionally, use of the PCO was validated in two different locations where Typhoid fever is endemic. The PCO was detected in all seeded field samples using all methods tested. Together, the lab and field experiments indicate that the PCO serves as a valid matrix spike to assess the efficacy of different ES methods.

## Methods

### Development and validation of process control organism

**Fig 1:**
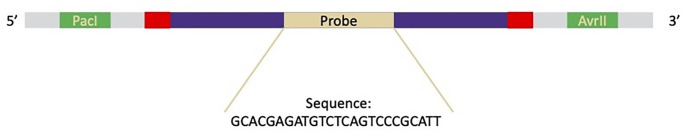
Modified amplicon structure of the process control organism. Random non-coding DNA (gray) surrounds the two inserted restriction sites (green). The primer sequences (red) are the original primer sequences. The target sequence of the qPCR assay (dark purple) contains the probe, random non-coding DNA (gold) that replaced the original probe sequence with a unique probe sequence to indicate the PCO. The total modified amplicon is 184 basepairs long.

The full protocol used to create the PCO is included in the supplemental material. Briefly, first a modified amplicon sequence was developed by amending the sequence for a commonly used *S*. Typhi assay targeting the *staG* gene (Fig 1) [19]. The *staG* probe sequence was replaced with a random, non-coding DNA sequence with restriction enzyme sites engineered at either end of the *staG* qPCR target. Additionally, because the qPCR target does not contain promoter sequences, there is no concern that this gene will be unintentionally transcribed in the PCO. Plasmid pGRG36 (Addgene plasmid #16666 http://n2t.net/addgene:16666; RRID:Addgene_16666) [20] and the modified amplicon were double digested with restriction enzymes following the NEBcloner protocol (New England Biolabs, Ipswich, MA, USA). The digested plasmid was then resolved with 0.8% agarose gel electrophoresis, and the band representing the digested plasmid was purified using the QIAquick gel extraction kit (QIAGEN Inc., Germantown, MD, USA). The modified amplicon and digested plasmid were then ligated together using the NEBElectroligase kit (New England Biolabs, Ipswich, MA, USA). Ligation was confirmed with conventional PCR using the leadpGRG36 primer targeting the plasmid and the *staG* reverse primer (Table 1). This product was then sequenced using primer pGRG36 [20].

**Table 1:** Primer and probe sequences for qPCR assays and sequencing.

Primer/Probe	Sequence (5’ → 3’)	Target	Ref.
**leadpGRG36**	GGGGTGGAAATGGAGTTTTT	pGRG36	McKenzie and Craig (18)
**Tn7-F**	GATGCTGGTGGCGAAGCTGT	tn7	
**Tn7-R**	GATGACGGTTTGTCACATGGA	tn7	
**staG-Frt**	CGCGAAGTCAGAGTCGACATAG	*S*. Typhi	Nga, Karkey (17)
**staG-Rrt**	AAGACCTCAACGCCGATCAC	*S*. Typhi	
**staG-Probe** ^a^	FAM-CATTTGTTCTGGAGCAGGCTGACGG-TAMRA	*S*. Typhi	
**STmod-Probe**	HEX-GCACGAGATGTCTCAGTCCCGCATT-BHQ	PCO	This manuscript
**ttr-F**	CTCACCAGGAGATTACAACATGG	Pan *Salmonella*	Nair, Patel (22)
**ttr-R**	AGCTCAGACCAAAAGTGACCATC	Pan *Salmonella*	
**ttr-Probe**	FAM-CACCGACGGCGAGACCGACTTT-BHQ1	Pan *Salmonella*	
**tviB-F**	TGTGGTAAAGGAACTCGGTAAA	*S*. Typhi	
**tviB-R**	GACTTCCGATACCGGGATAATG	*S*. Typhi	
**tviB-Probe**	TET-TGGATGCCGAAGAGGTAAGACGAGA-BHQ1	*S*. Typhi	

^a^ The LSTM *staG* probe contained Cy5 on the 5’ end rather than FAM.

**Fig 2:**
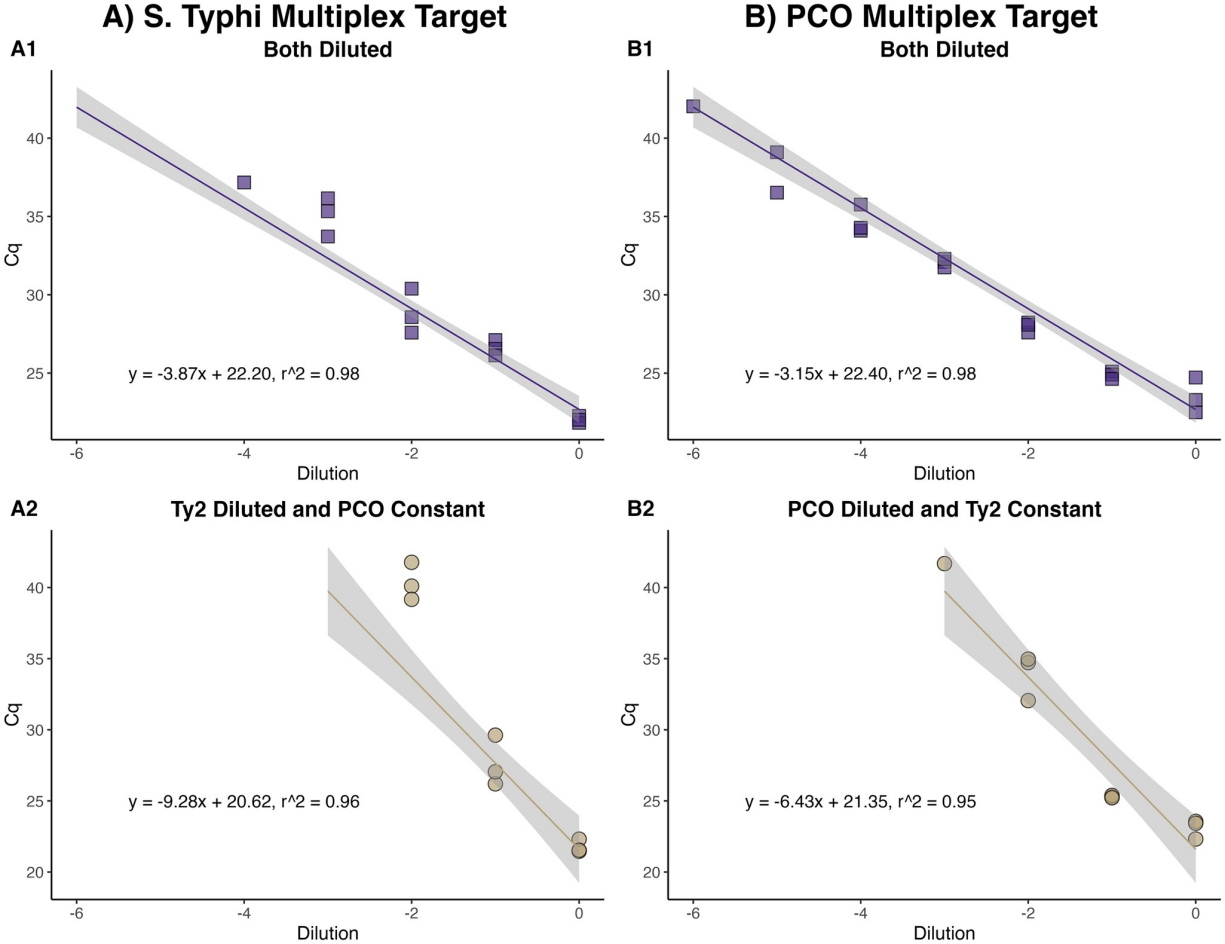
Standard curves of serial ten-fold dilutions in nuclease-free water. A) *S*. Typhi and B) the process control organism (PCO) in comparison to different concentrations of the opposite target in multiplex qPCR reactions. Each concentration was run in triplicate. When one target was held constant at a single concentration but the other diluted (A2, B2), the slope of the diluted target increases, suggesting the higher concentrated target is outcompeting for the primers.

The ligated plasmid was transformed into TOP10 *E*. *coli* host cells (ThermoFisher Scientific, Inc., Waltham, MA, USA). Bacteria were grown overnight in LB broth at 32°C with 25 μg/mL of ampicillin to select for transformants. Next, the Tn7 insertion mechanism contained on pGRG36 was induced using LB ampicillin broth containing 5% arabinose. Overnight cultures were then plated and grown at 42°C to block replication of the plasmid [20]. Conventional PCR was carried out on 12 colonies using primers flanking the Tn7 attachment site in the bacterial genome (Table 1). The PCR products were run on an agarose gel. Colonies containing the insertion produced a product of 1,738 bp as compared to 678 bp for colonies without the insertion Fig 2 in S1 File). Bacterial clones which produced the 1,738 bp amplicon were then sequenced with Sanger sequencing using the forward Tn7 primer targeting the genome (Table 1).

### DNA extraction and analysis

DNA extraction was performed on the samples using the QIAamp PowerFecal Pro DNA Kit (Qiagen, Hilden, Germany) with the following modifications to the manufacturer’s instructions. The input material was either pelleted 1 mL aliquots of the samples or sliced membrane filters, as described in the supplemental information, and the DNA was eluted in 120 μL of solution C6.

### qPCR assay

A commonly used qPCR assay to detect *S*. Typhi in clinical samples was altered to detect the developed process control organism (PCO) and *S*. Typhi [19]. The reaction was performed using iTaq Universal Probes Supermix (Bio-Rad Laboratories, Inc., Hercules, CA, USA). The forward and reverse primers for *staG* were used to amplify both the PCO and *S*. Typhi but with differentially labeled probes specifically targeting each organism (Table 1). To optimize the primer concentrations, reactions were run with primer concentrations of 0.4μM, 0.6μM, and 0.8μM in either SYBR green (iTaq Universal SYBR Green Supermix, Bio-Rad) or with both probes at a concentration of 0.15μM. All reactions were run with standard curves of purified DNA from both PCO and *S*. Typhi in molecular grade water. Once an optimum primer concentration was determined, the reactions were run with standard curves diluted in a wastewater extract to control for the matrix effect. Samples were run in duplicate or triplicate and included undiluted and 10-fold dilutions. Molecular grade water was the no template control (NTC).

### Organism culture and enumeration

Ty2, a commonly used pathogenic strain of *S*. Typhi was utilized as a positive control in this study. We confirmed Ty2 was positive for the Vi antigen using a Vi antigen agglutination test [21]. Ty2 was grown in the dark using LB-Miller broth with a supplemental aromatic amino acid mix and 50 ng/mL ferrioxamine E (Millipore, Burlington, MA, USA) as previously described [21]. The PCO was grown using LB Miller broth. PCO and Ty2 inocula for experiments were prepared by growing the organisms for a specified period of time at 37°C, harvesting them during exponential growth phase, and storing single-use aliquots of the cultures in 30% glycerol until use at -80°C. Prior to planned experiments, 20 μL of the frozen Ty2 and PCO glycerol stocks were inoculated into separate conical flasks with 15 mL of liquid media and incubated with shaking (200 rpm, 37°C, 12–16 hours). To determine the colony forming units (CFUs) of the organisms seeded, spread plates with 100 μL of relevant dilutions were plated and incubated overnight at 37°C.

### Lab-based seeded methods

Influent wastewater (after bar screens) grab samples were collected from a local wastewater treatment plant in Seattle, WA, USA. The sample collection and analysis plan was approved by the King County Wastewater Treatment Division Research Coordinator prior to the start of the study. Grab samples were stored at 4°C until processing (up to 3 days). Varying concentrations of the PCO were seeded into 10 mL of 1x phosphate buffered saline (PBS, pH = 7.2), vortexed (30 seconds), and dosed into the wastewater. Additionally, Ty2 was seeded into 10 mL of 1x PBS, vortexed (30 seconds), and dosed into the wastewater. The final concentration of the PCO and Ty2 in the seeded wastewater varied by experiment (Table 2). The seeded wastewater was thoroughly mixed and distributed for processing by filter cartridge [22,23], recirculating Moore swab [24,25], membrane filtration [25], and differential centrifugation methods using a peristaltic pump while continuously shaking following methods previously published [21]. Methods were chosen after discussions with partners in the Typhoid Environmental Surveillance Working Group and were adapted to the Environmental and Occupational Health Microbiology Lab after site visits to partner labs. A complete description of these methods and the volumes assayed is in the supplemental information Table 1 in S1 File). Samples were extracted and analyzed for Ty2 and the PCO as described above, using a DNA input volume of 5 μL.

**Table 2:** Expected concentrations of PCO and Ty2 seeded in wastewater for experiments conducted in Seattle, WA.

Methods	PCO seeded (CFU/mL)	Ty2 seeded (CFU/mL)	*n*
**Filter cartridge**	1	0.1	6
	10	0.1	11
	10	1	2
	10	10	3
	100	1	2
**Membrane filtration**	1	0.1	6
	10	0.1	11
	10	1	2
	10	10	3
	100	1	2
**Recirculating Moore Swab**	1	0.1	6
	10	0.1	11
	10	1	2
	10	10	3
	100	1	2
**Differential centrifugation**	1	0.1	3
	10	0.1	3

### Field-based seeded methods

Use of the PCO during *S*. Typhi ES was also assessed at two locations with endemic Typhoid: Vellore, India and Blantyre, Malawi. In Vellore, permission to conduct environmental surveillance was obtained from the concerned Vellore city Corporation official and the elected political representative of the study area. In Blantyre, activities were approved by an ethics waiver from the University of Malawi College of Medicine Research Ethics Committee and by the Blantyre City Council. Additional information regarding the ethical, cultural, and scientific considerations specific to inclusivity in global research is included in the Supporting Information (SI Checklist 1). The methods utilized included filter cartridges, recirculating Moore swabs, and membrane filtration in Vellore, and field Moore swabs and membrane filtration in Malawi (Table 3). Four sampling sites in Vellore were chosen Table 2 in S1 File), with three replicates per method collected per site, for a total of 36 samples (Table 3). All sites were open channel systems. Samples were collected using sterile collection bottles and then seeded with approximately 10 CFU/mL of the PCO prior to returning to the lab, determined by spread plating on LB agar. Filter cartridge samples were filtered in the field. The filtered volumes are summarized in SI Table 3 in S1 File. After sampling, the cartridge filters and six liters of wastewater (1L for membrane filtration, 5L for Moore swabs) were transported to the laboratory for processing and analysis. After processing, the samples were extracted using the Qiagen QIAamp PowerFecal Pro DNA Kit as described above. DNA extracts were analyzed for *staG* and the PCO targets using the qPCR assay described above.

**Table 3:** Number of replicates for each method at each location.

Location	Sites	Methods and Replicates per Sampling Time				Timepoints per site	*n*
		Filter cartridge	Recirc. Moore swab	Field Moore swab	Membrane filtration		
Vellore	4	3	3	0	3	1	36
Blantyre	3	0	0	3	3	2	36

Three sampling sites in Blantyre were chosen (SI Table 2 in S1 File), with three replicates per method collected per site at two time points, for a planned total of 36 samples (Table 3). One liter membrane filtration samples were transported to the laboratory for seeding with approximately 1 CFU/mL of the PCO, processing, and analysis. A minimum volume of 500 mL was filtered. Field Moore swabs were placed in the water flow at each site, collected after 48 hours, and transported to the laboratory for processing. Of the 18 placed Moore swabs, seven were lost for unknown reasons. After processing, the samples were extracted using the Qiagen QIAamp PowerFecal Pro DNA Kit and eluted into 100 μL. DNA extracts were analyzed for the *staG*, *tviB*, and *ttr* genes using a previously published assay [26], as well as the PCO using a singleplex version of the assay described in the qPCR Assay section of the Methods. The type of *Salmonella* present in the samples was predicted using the targets detected, with positive *staG*, *tviB* and *ttr* yielding *S*. Typhi; *tviB* and *ttr* yielding presumptively positive *S*. Typhi; *staG* and *ttr* yielding presumptively positive non-typhoidal salmonella (NTS); and *ttr* yielding NTS (SI Table 4 in S1 File).

### Data analysis

All qPCR data were analyzed using Bio-Rad CFX Maestro for Mac (Bio-Rad Laboratories, Hercules, CA, USA), and data were collated and managed using Microsoft Excel (Microsoft Corp., Redmond, WA, USA). Cycle thresholds were manually set at the point where positive control standard curves started exponentially multiplying. Data were further analyzed, and figures were generated using RStudio (Posit team (2023). RStudio: Integrated Development Environment for R. Posit Software, PBC, Boston, MA. URL http://www.posit.co/). and associated packages.

## Results and discussion

### Sequencing, qPCR

To confirm that the modified amplicon (Fig 1) inserted correctly into the *E*. *coli* genome, conventional PCR followed by gel electrophoresis was run on randomly selected colonies. Five colonies were found to contain the correct amplicon length of 1738 bp (SI Fig 2 in S1 File). Sanger sequencing was carried out on the amplicons using the Tn7 primer targeting the bacterial genome to confirm the modified amplicon was inserted in the correct location and orientation (Table 1). Standard curves for the newly designed PCO and *S*. Typhi multiplexed PCR assay were then run in triplicate under three different reaction conditions:

1. 1:10 serial dilutions of the modified PCO with *S*. Typhi at a constant concentration of 0.2ng/μL,
2. 1:10 serial dilutions of *S*. Typhi with the modified PCO at a constant concentration of 0.2ng/μL,
3. 1:10 serial dilutions of both *S*. Typhi and the modified PCO beginning with a concentration of 0.2ng/μL each.

To confirm that there was no cross reactivity between the probes, singleplex assays were run for the PCO and *staG* using the above standard curves. No cross reactivity between either probe was detected, indicating the PCO has utility in *S*. Typhi ES. Next, different concentrations of primers were added to assess the optimal primer concentration for the multiplex assay (0.4μM, 0.6μM, 0.8μM, and 1.0μM). The reaction efficiency decreased as the primer concentration increased, with 0.4μM selected as the optimal primer concentration for future applications.

As both targets use the same primer set, there were interactions between the genetic targets in the multiplex assay. When one target was maintained at a single concentration and the other target serially diluted, the slope of the standard curve increased (Fig 2). This suggests that the more highly concentrated target interferes with amplification of the lower concentrated target due to competition for the primers and other key reagents for DNA amplification. This target interference indicates the PCO would be most useful as a matrix spike to understand *S*. Typhi recovery and the level of inhibition in a given sample, rather than as a seeded control when trying to enumerate low or unknown levels of *S*. Typhi. Matrix spikes are used to identify method performance and inhibition associated with a new sample type or sampling location and should be used every 20 samples to understand performance over time (25). When used as a seeded recovery control for *S*. Typhi ES, samples should be processed using a split-seed approach where a sample is collected, split into two, and one of the aliquots is seeded with the PCO. Furthermore, because the modified gene is integrated into the genome of the PCO, the PCO can be added to samples collected from the environment without fear of loss of the plasmid. As wastewater surveillance continues to expand, there is a need for validated recovery control organisms. The PCO could potentially be utilized as a seeded recovery control for ES of other gram-negative bacteria.

### Laboratory seeded studies

**Fig 3:**
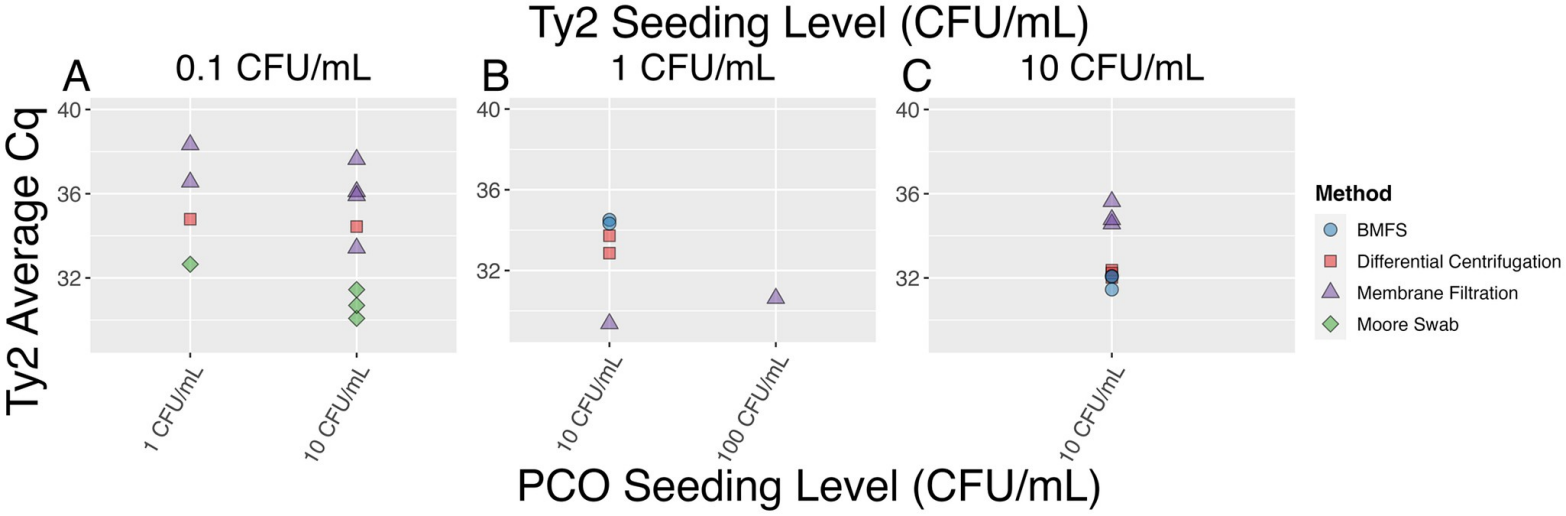
Ty2 Cq faceted by expected Ty2 seeding level. A) 0.1 CFU/mL, B) 1 CFU/mL, and C) 10 CFU/mL and expected process control organism (PCO) seeding level (CFU/mL). There is higher variability in Cq within a single method for Ty2 when it is seeded at lower levels relative to the PCO.

To test the applicability of utilizing the PCO as a matrix spike, wastewater from a local Seattle-area treatment plant was seeded with the PCO and Ty2 and concentrated using four different methods: filter cartridge, recirculating Moore swab, membrane filtration, and differential centrifugation. The PCO was detected in all samples of all four methods with expected seeding levels of 10 or 100 CFU/mL and was detected in 66.7% of samples or higher for each method seeded with 1 CFU/mL (Table 4). Similarly, Ty2 was detected in all samples of all methods tested with an expected seeding level of 10 CFU/mL (Table 5). Ty2 was detected in 50% of samples for all methods tested when seeded with approximately 1 CFU/mL (Table 5). Furthermore, when Ty2 was seeded at 10 CFU/mL, relatively high for what would be expected in the environment, and the PCO was seeded at 10 CFU/mL, Ty2 was detected with all methods and replicates at the expected Cq values (Fig 3). Notably, the Ty2 Cq variability within each method was lower when it was seeded at the same concentration as the PCO (10 CFU/mL), compared to when it was seeded at a lower concentration as the PCO (Fig 3). The PCO performed similarly in seeded lab studies across methods and seeding concentrations while still allowing for *S*. Typhi detection, indicating it serves as a viable model organism for *S*. Typhi.

**Table 4:** Average Cq and percent detection of the PCO in seeded lab studies at different concentrations. The PCO was detected in all experiments when it was seeded at 10 CFU/mL or higher and was detected in all experiments when it was seeded at 1 CFU/mL for filter cartridge and differential centrifugation methods.

	1 CFU PCO/mL		10 CFU PCO/mL		100 CFU PCO/mL	
	Positive Cq<40	Cq average	Positive Cq<40	Cq average	Positive Cq<40	Cq average
Membrane filtration	4/6 (66.7%)	37.9	16/16 (100%)	33.1	2/2 (100%)	30.3
Filter cartridge	6/6 (100%)	35.5	16/16 (100%)	31.5	2/2 (100%)	28.7
Recirculating Moore swab	4/6 (66.7%)	37.7	16/16 (100%)	32.7	2/2 (100%)	29.8
Differential centrifugation	3/3 (100%)	36.2	3/3 (100%)	32.6	-	-

**Table 5:** Average Cq and percent detection of Ty2 in seeded lab studies at different concentrations. Ty2 was detected in all experiments when it was seeded at 10 CFU/mL and 50% of all experiments when it was seeded at 1 CFU/mL. There was less detection when it was seeded at 0.1 CFU/mL, but this is likely due to a wider variability in actual seeding level at lower concentrations.

	0.1 CFU Ty2/mL		1 CFU Ty2/mL		10 CFU Ty2/mL	
	Positive Cq<40	Cq average	Positive Cq<40	Cq average	Positive Cq<40	Cq average
Membrane filtration	6/17 (35.3%)	35.7	2/4 (50%)	33.3	3/3 (100%)	32.2
Filter cartridge	0/17 (0%)	N/A	2/4 (50%)	34.4	3/3 (100%)	31.9
Recirculating Moore swab	3/14 (21.4%)	30.7	2/4 (50%)	30.0	3/3 (100%)	35.0
Differential centrifugation	2/3 (66.7%)	34.4	Not tested		Not tested	

**Fig 4:**
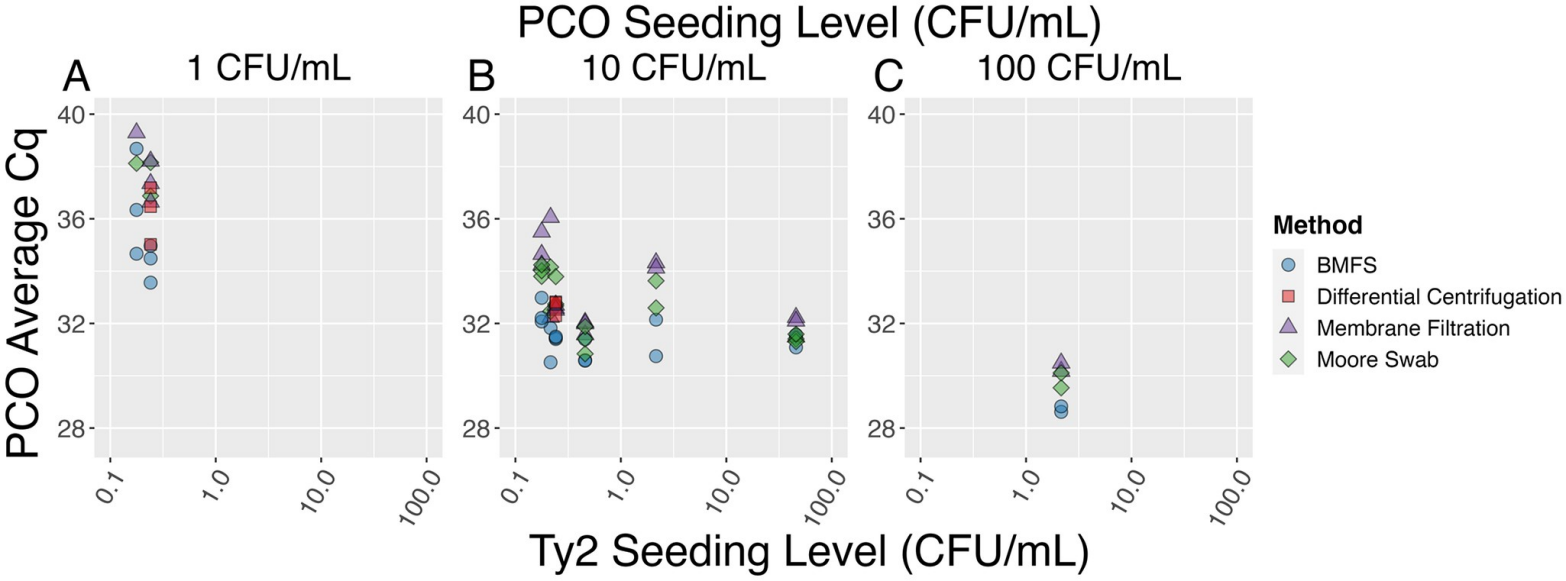
PCO Cq faceted by expected PCO seeding level (CFU/mL). A) 1 CFU/mL, B) 10 CFU/mL, and C) 100 CFU/mL and actual Ty2 seeding level (CFU/mL). When the PCO is seeded at higher concentrations relative to Ty2, there is more variability in Cq, as is seen in B.

Because the qPCR reaction efficiency decreases when the concentrations of the two bacteria become more dissimilar (Fig 2), the ideal seeding level of the PCO requires knowledge of the background levels of *S*. Typhi that would be expected in a sample. This is readily apparent when assessing the PCO Cq by organism seeding level (Fig 4). When the PCO is seeded at a higher relative concentration compared to Ty2, there is greater variability in the Ty2 results compared to when they are seeded at similar concentrations (Fig 4). To utilize the PCO as a spike-in matrix control when sampling at a new location or with a new method, the PCO must be seeded at a high enough concentration to not be subject to interference by Ty2.

Due to the qPCR interference of the intended target, the PCO should be used as a matrix spike and not as a routine recovery control organism. Prior to the development of the PCO, there was no matrix spike to conduct QA/QC for *S*. Typhi ES. Due to its similar performance in laboratory methods being used for *S*. Typhi wastewater surveillance, the PCO serves as a viable matrix spike to meet QA/QC standards. The PCO can help establish that methods work as expected in new settings or are continuing to work as expected when seeded every few weeks.

### In-country application of the PCO

Use of the PCO as a matrix spike was assessed in samples collected from two Typhoid endemic areas: Vellore and Blantyre. All filter cartridges, membrane filtration, and recirculating Moore swabs samples in Vellore were positive for the PCO (Table 6). The PCO Cq values ranged from 30.8 to 39.5, with 86% of samples being less than 35.0 Cq. The Cq values were similar across all sites and timepoints (Table 6). Additionally, the Cq values were similar across methods with an average (± standard deviation) of 32.9 ±1.03 for filter cartridge, 34.0 ±1.03 for membrane filtration, and 33.3 ±2.54 for recirculating Moore swabs. All membrane filtration samples in Blantyre were positive for the PCO (Table 6). The PCO Cq values ranged from 17.7 to 27.9, with all samples less than 35.0 Cq.

**Table 6:** PCO Cq values in field experiments from seeding with 10 CFU/mL in Vellore, India and 1 CFU/mL in Blantyre, Malawi, and S. Typhi Cq targets.

		Date (dd/ mm/yyyy)	Method	PCO		*staG*		*tviB*	
				Mean Cq (min, max)	<40 Cq positive	Mean Cq (min, max)	<40 Cq positive	Mean Cq (min, max)	<40 Cq positive
Vellore, India	Site 1	27/01/2020	Moore swab*	31.9 (31.4, 32.7)	3/3	neg	0/3	N/A	N/A
		27/01/2020	Mem. filtration	33.8 (33.5, 34.1)	3/3	neg	0/3	N/A	N/A
		27/01/2020	BMFS	33.0 (32.2, 34.1)	3/3	neg	0/3	N/A	N/A
	Site 2	28/01/2020	Moore swab*	32.0 (31.2, 32.4)	3/3	35.2 (34.7, 35.7)	3/3	N/A	N/A
		28/01/2020	Mem. filtration	33.2 (31.8, 34.1)	3/3	36.4	1/3	N/A	N/A
		28/01/2020	BMFS	32.3 (31.6, 33.0)	3/3	39.8	1/3	N/A	N/A
	Site 3	29/01/2020	Moore swab*	32.6 (30.8, 34.0)	3/3	36.2 (34.2, 38.4)	3/3	N/A	N/A
		29/01/2020	Mem. filtration	35.3 (34.7, 35.7)	3/3	neg	0/3	N/A	N/A
		29/01/2020	BMFS	34.0 (33.3, 35.1)	3/3	neg	0/3	N/A	N/A
	Site 4	30/01/2020	Moore swab*	36.9 (34.4, 39.5)	3/3	35.5 (34.6, 36.2)	3/3	N/A	N/A
		30/01/2020	Mem. filtration	33.8 (33.2, 34.3)	3/3	neg	0/3	N/A	N/A
		30/01/2020	BMFS	32.2 (31.8, 33.0)	3/3	neg	0/3	N/A	N/A
Blantyre, Malawi	Site 1 (Manase)	27/01/2021	Moore swab†	N/A	N/A	29.9 (29.6, 30.3)	2/3	30.8	1/3
		27/01/2021	Mem. Filtration	22.2 (19.0, 24.9)	3/3	neg	0/3	30.9	1/3
		10/02/2021	Moore swab	N/A	N/A	30.7 (29.5, 32.4)	2/2 ‡	33.7	1/2 ‡
		10/02/2021	Mem. filtration	21.9 (18.6, 26.4)	3/3	neg	0/3	33.0 (31.6, 34.5)	2/3
	Site 2 (Mbayani)	27/01/2021	Moore swab†	N/A	N/A	29.8 (29.0, 30.2)	2/2 ‡	Neg	0/2‡
		27/01/2021	Mem. filtration	24.2 (21.7, 27.9)	3/3	neg	0/3	Neg	0/3
		10/02/2021	Moore swab	N/A	N/A	33.0 (32.9, 33.2)	2/2 ‡	Neg	0/2 ‡
		10/02/2021	Mem. filtration	19.5 (18.6, 20.2)	3/3	neg	0/3	Neg	0/3
	Site 3 (Ndirande)	27/01/2021	Moore swab†	N/A	N/A	30.8	1/1 §	Neg	0/1 §
		27/01/2021	Mem. filtration	20.0 (18.9, 21.3)	3/3	neg	0/3	Neg	0/3
		10/02/2021	Moore swab	N/A	N/A	33.2	1/1 §	Neg	0/1 §
		10/02/2021	Mem. filtration	21.2 (20.2, 22.4)	3/3	neg	0/3	Neg	0/3

* Recirculating Moore swab.

^†^ Field Moore swab.

^‡^ One replicate Moore swab lost.

^§^ Two replicate Moore swabs lost.

The PCO Cq values seeded at 10 CFU/mL for both the lab studies and the Vellore, India field study are very similar (Tables 4 and 6). The wastewater from these sampling locations includes wastewater treatment plants in large and economically diverse population centers, open water channels in urban and rural centers, and natural waterways (SI Table 2 in S1 File). This suggests that the PCO performs similarly in vastly different water matrices. This versatility is crucial for an effective enable QA/QC organism. However, when seeded at 1 CFU/mL in Seattle and Blantyre the mean Cq values are not similar (Tables 4 and 6). This could be due to differences in when the seeding occurred or in actual seeding levels, as the seeding levels in Seattle were lower than expected (SI Fig 1 in S1 File). There could also be substantially fewer inhibitors in the Blantyre water samples compared to Seattle samples, as inhibitors have been shown to negatively affect detection via qPCR [27]. Wastewater samples collected in Seattle likely had more inhibition than fecally impacted surface water samples collected in Blantyre because wastewater has been shown to have more PCR inhibition than other water samples [28]. These differences in detection at the same seeding level highlight its proper use as a matrix spike and not a regular recovery control organism for *S*. Typhi ES. While changes in detection at a single location would require investigation, differences between matrices is expected and does not necessarily indicate poor performance [29,30].

In Vellore, all recirculating Moore swab samples at Sites 2, 3 and 4 were positive for *staG* (Cq <40) (Table 6). One filter cartridge and one membrane filtration sample at Site 2 were positive for *staG*. In Blantyre, ten of the eleven recovered field Moore swab samples were positive for *staG* with detection at all three sites, while no membrane filtration samples were positive for *staG*. Additionally, two field Moore swab and three membrane filtration samples were positive for *tviB*, all from Site 1. These results suggest three membrane filtration samples were presumptively positive for *S*. Typhi *(tviB* and *ttr* positive) and one was positive for NTS *(ttr* positive), while two field Moore swab samples were positive for *S*. Typhi *(staG*, *tviB*, and *ttr* positive); one positive for NTS *(ttr* positive); and eight presumptively positive for NTS *(staG* and *ttr* positive). For the two field Moore swab samples that were positive for *S*. Typhi, the matching membrane filtration samples were presumptively positive for *S*. Typhi.

### Limitations

Primary limitations of this study include precision in the seeding levels of the PCO and *S*. Typhi and the background matrix. Because the desired seeding level relied on dilutions of overnight cultures, the amount that was seeded for both Ty2 and the PCO was not always equal to what was expected (SI Fig 1 in S1 File). This suggests that some variability in results could be due to the seeding level and not the methods themselves. Additionally, laboratory based seeding experiments were conducted over a few months. This adds variability between experimental matrices, in addition to variability between Seattle, USA, Vellore, India and Blantyre, Malawi. However, the success of the PCO across experimental matrices suggests it performs well despite these differences. Furthermore, because detection methods using a *S*. Typhi enrichment step that inhibits coliform growth, such as selenite broth [31], may also prevent growth of the PCO, the PCO will have limited utility as a quantitative control in these methods, particularly at low seeding levels. However, the PCO was detectable using the recirculating Moore Swab method (Table 4), suggesting it is applicable with certain *S*. Typhi enrichment broths. An additional limitation of the multiplex qPCR is the interference between the two gene targets when they are at exponentially different concentrations. While this behavior is to be expected, it does not negate the use of the PCO in *S*. Typhi ES.

## Conclusions

This study indicates the PCO performs similarly to *S*. Typhi in a variety of water matrices at different times and with different methods. Feedback from international sampling and processing teams suggest the PCO is easy to grow and use because it is non-pathogenic, does not require special growth media or temperatures and can be readily transported. Because it is a BLS1 organism, there are fewer import and laboratory regulations to apply it in a new country. Additionally, being an *E*. *coli* K-12 strain, it cannot present or persist as a bio- or environmental hazard. Because it is non-pathogenic, the PCO is more applicable than a pathogenic *S*. Typhi as a seeded control. Due to its similar performance in methods being used for *S*. Typhi wastewater surveillance, the PCO serves as a viable matrix spike to meet QA/QC standards and to identify inhibition in a new sampling location or given sample. It can also be used as a seeded method recovery control organism when *S*. Typhi concentrations are low or unknown if a split-seed approach is used. Finally, the PCO can be used as a method recovery control organism for ES of other gram-negative bacteria.

## Supporting information

S1 File SI Table 1: Volumes added and effective volume assayed for each experimental method. SI Fig 1: Percent difference of actual seeding level compared to what was expected for both A) Ty2 and B) the PCO. SI Table 2: Qualitative description of sampling sites and GPS locations. SI Table 3: Sample volumes filtered in Vellore, India. SI Table 4: The combination of gene targets needed to be positive for a sample to be considered positive for that organism. Positive results for all three gene targets are needed for *S*. Typhi, but a non-typhoidal salmonella (NTS) is only positive for *ttr*. SI Fig 2: Gel image of the PCR targeting the Tn7 region of the genome. Lanes containing 1738 bp amplicons indicate bacteria that have the insertion into the genome, while lanes with 678 bp amplicons do not have the insertion.
(DOCX)

S1 Data (XLSX)

S1 Raw images (PDF)
