# Supplementary material for: Development, confirmation, and application of a seeded Escherichia coli process control organism to validate Salmonella enterica serovar Typhi environmental surveillance methods
Source: PLoS One. 2024 May 7;19(5):e0301624. doi: 10.1371/journal.pone.0301624 (PMC11075847; doi:10.1371/journal.pone.0301624)
Supplement: S1 File — SI Table 1: Volumes added and effective volume assayed for each experimental method. SI Fig 1: Percent difference of actual seeding level compared to what was expected for both A) Ty2 and B) the PCO. SI Table 2: Qualitative description of sampling sites and GPS locations. SI Table 3: Sample volumes filtered in Vellore, India. SI Table 4: The combination of gene targets needed to be positive for a sample to be considered positive for that organism. Positive results for all three gene targets are needed for S. Typhi, but a non-typhoidal salmonella (NTS) is only positive for ttr. SI Fig 2: Gel image of the PCR targeting the Tn7 region of the genome. Lanes containing 1738 bp amplicons indicate bacteria that have the insertion into the genome, while lanes with 678 bp amplicons do not have the insertion. (DOCX) [file pone.0301624.s001.docx]

# **Supplemental Methods**

## **Development and Validation of Process Control Organism**

Plasmid pGRG36 was obtained from Addgene and was a gift from Nancy Craig (Addgene plasmid # 16666 ; http://n2t.net/addgene:16666 ; RRID:Addgene_16666) (1). The plasmid was amplified and isolated using the Invitrogen ChargeSwitch-Pro Plasmid Miniprep kit (Thermo Fisher Scientific, Waltham, MA). The plasmid was digested using the restriction enzymes AvrII and PacI in 10x NEBuffer 3.1 (New England Biolabs, Ipswich, MA). The reaction was carried out following the NEBcloner protocol: incubation at 37°C for 5-15 minutes and heat inactivation at 65°C for 20 minutes. The reaction mix contained 5.0uL of 10x NEBuffer 3.1, 1uL each of PacI and AvrII, 1ug of plasmid DNA, and water up to 50uL. The double-digested plasmid was then run on a 0.8% agarose gel and purified using the QIAquick gel extraction kit (QIAGEN Inc., Germantown, MD).

The amplicon sequence for insertion into the digested plasmid was created by modifying the target sequence for a commonly used *S.* Typhi qPCR assay (2). The original probe sequence was removed and replaced with a sequence of random, non-coding bases. To either end of the target sequence, PacI (5’ end) and AvrII (3’ end) restriction sites were added with non-coding DNA (Fig 1). The sequence was digested using the same digestion protocol as the plasmid digestion.

The digested modified amplicon sequence was ligated into the digested plasmid using the NEB Electroligase kit (New England Biolabs, Ipswich, MA). The reaction contained 20-100 nanograms of plasmid with a 3-fold molar excess of insert. The reaction was carried out in 5uL of ElectroLigase Reaction buffer with 1uL of the ElectroLigase enzyme. It was incubated at 25°C for one hour and then inactivated at 65°C for 15 minutes. Ligation was confirmed with conventional PCR using the leadpGRG36 primer targeting the plasmid and the *S.* Typhi qPCR assay reverse primer (Table 1). This product was then sequenced using primer pGRG36.

The ligated plasmid was transformed into top10 *E. coli*. Bacteria were grown overnight in LB broth at 32°C with 25 ug/mL of ampicillin to select for bacteria containing the plasmid. Insertion into the genome was carried out by transferring the overnight liquid culture to fresh LB broth containing 25ug/mL of ampicillin and inducing the Tn7 insertion mechanism with 5% arabinose (1). Bacteria were grown at 32°C and 225rpm for seven and a half hours before the culture was diluted and plated onto LB plates for overnight growth. Overnight plates were grown at 42°C to block replication of the plasmid. Individual colonies from these plates were re-streaked onto fresh LB plates and grown at 42°C to ensure loss of the plasmid.

Twelve individual colonies were selected for further confirmation analysis. They were grown overnight in LB broth at 37°C with 225rpm. DNA extraction was carried out following the DNA purification from Blood or Body Fluids spin protocol from the QIAamp DNA Mini Kit (QIAGEN Inc., Germantown, MD) with the following adjustments: spun down 1mL of the overnight culture, resuspended the pellet in 200uL of 1% PBS, add 20uL proteinase K before continuing with step 3. Conventional PCR was carried out on the DNA extracts using primers flanking the Tn7 attachment site in the bacterial genome (Table 1). The PCR product was run on a gel and bacteria containing the insertion were 1738bp compared to 678 bp. Bacteria with the correct length of DNA were then sequenced using the forward Tn7 primer targeting the genome (Table 1).

## **Lab-Based Seeded Methods**

### **Filter cartridge samples**

Seeded wastewater was filtered through 2” ViroCap filters for 40 minutes or until 3 L had passed, whichever is longer according to Zhou, Ong (3). An average of 4.4±0.49 L (*n*=24; 95% CI) were filtered, resulting in 46 mL of the original sample entering the qPCR assay (SI Table 1). The filters were eluted via a double elution with a 1.5% beef extract, 0.05 M glycine solution, pH 9.5. Skimmed milk flocculation was performed as previously described with the pellets resuspended in a total of 4-mL 1x PBS (4). 1-mL aliquots of the resuspended sample were then centrifuged (10,000xg, 10 minutes), the supernatant discarded, and stored at -20°C for DNA extraction.

### **Moore swab samples**

Moore swabs were made by accordion-folding sterile hospital gauze and tying a nylon string in the middle. The Moore swabs were placed in 10-L carboys containing 5 L of the seeded wastewater, which was recirculated using a peristaltic pump a slow rate to simulate laminar flow in sewage conveyance line or river system. The seeded wastewater was recirculated for 24 hours, followed by removing the swabs and enriching them overnight in Universal Pre-enrichment (UPE) broth at 37°C. Membrane filtration was performed on 20-mL of the enrichment. The membrane filter was sliced into 6-10 pieces, placed in a 2 mL tube suitable for bead beating, and stored at -20°C until DNA extraction.

### **Membrane filtration samples**

For processing, the seeded wastewater was added to a coffee pre-filter placed on top of the membrane filter cup. Due to filter clogging, the sample was filtered for 1 hour, then the unfiltered volume on the coffee filter and the membrane filter were removed and the filters replaced. Up to 5 coffee filters and membrane filters were used per sample. The final unfiltered volume was recorded. An average of 496±46 mL (*n*=23; 95% CI) was filtered, resulting in 2.1 mL of the original sample entering the qPCR assay (SI Table 1). The 5 membrane filters were transferred to a WhirlPak bag and 10 mL Ringer’s lactate was added. The filter discs were massaged until they appeared clean or broke apart. 1-mL aliquots of the eluate was then centrifuged (10,000xg, 10 minutes), the supernatant discarded, and stored at -20°C for DNA extraction.

### **Differential centrifugation samples**

Samples were processed by centrifugation in 50-mL conicals (1 minute, 1000 rpm, 4°C). One-hundred (100) mL volumes were processed, resulting in 2.1 mL of the original sample entering the qPCR assay (SI Table 1). The supernatant was transferred to fresh 50-mL conicals and centrifuged (15 minutes, 4000 rpm, 4°C). The supernatant was discarded and the pellets resuspended in a total of 2-mL 1x PBS. 1-mL aliquots of the resuspended sample was then centrifuged (10,000xg, 10 minutes), the supernatant discarded, and stored at -20°C for DNA extraction.

# **Supplemental Data**

**SI Table 1: Volumes added and effective volume assayed for each experimental method.**

| Methods | Initial volume | Average processed volume | Final volume | Enrichment | Effective volume assayed |
| --- | --- | --- | --- | --- | --- |
| Filter cartridge | 6 L | 4.4 L | 4 mL | N/A | 46 mL |
| Membrane filtration | 1 L | 496 mL | 10 mL | N/A | 2.1 mL |
| Moore swab | 5 L | N/A | N/A | UPE | N/A |
| Differential centrifugation | 100 mL | 100 mL | 2 mL | N/A | 2.1 mL |

| **A)** | **B)** |
| --- | --- |
| 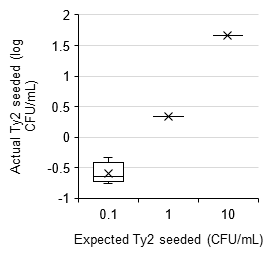 | 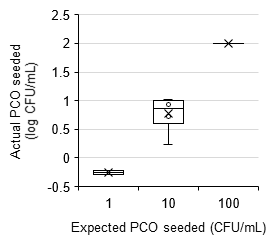 |

**SI Fig 1: Percent difference of actual seeding level compared to what was expected** for both A) Ty2 and B) the PCO.

**SI Table 2: Qualitative description of sampling sites and GPS locations**

|  | GPS coordinates | Information about site |
| --- | --- | --- |
| Vellore, India | TBD | Sathuvacheri, open channel system (small channel entering large river) |
|  | TBD | Vspm, open channel system (small creek like, greenery) |
|  | TBD | Rnp, open channel system (densely populated neighborhood) |
|  | TBD | SSK Maniyam, open channel system (neighborhood by a temple) |
| Blantyre, Malawi | TBD | Treatment plant in Manase that flows into the heavily contaminated Mudi River |
|  | TBD | A small water channel in the densely populated aread of Mbayani with markets and housing |
|  | TBD | The Nasolo River in the densely populated Ndirande residential area |

**SI Table 3: Sample volumes filtered in Vellore, India**

|  | Sample | Volume filtered (L) |  | Sample | Volume filtered (mL) |
| --- | --- | --- | --- | --- | --- |
| Site 1 | BMFS 1 | 3.4 |  | Membrane filtration 1 | 490 |
|  | BMFS 2 | 5.7 |  | Membrane filtration 2 | 540 |
|  | BMFS 3 | 4.9 |  | Membrane filtration 3 | 535 |
| Site 2 | BMFS 1 | 4.4 |  | Membrane filtration 1 | 600 |
|  | BMFS 2 | 5.5 |  | Membrane filtration 2 | 630 |
|  | BMFS 3 | 5.2 |  | Membrane filtration 3 | 670 |
| Site 3 | BMFS 1 | 3.0 |  | Membrane filtration 1 | 320 |
|  | BMFS 2 | 3.0 |  | Membrane filtration 2 | 330 |
|  | BMFS 3 | 5.0 |  | Membrane filtration 3 | 310 |
| Site 4 | BMFS 1 | 4.0 |  | Membrane filtration 1 | 390 |
|  | BMFS 2 | 4.5 |  | Membrane filtration 2 | 370 |
|  | BMFS 3 | 4.2 |  | Membrane filtration 3 | 410 |

**SI Table 4: The combination of gene targets needed to be positive for a sample to be considered positive for that organism.** Positive results for all three gene targets is needed for *S.* Typhi, but a non-typhoidal salmonella (NTS) is only positive for *ttr*.

| Gene target | *S.* Typhi | Presumptive *S.* Typhi | Presumptive NTS | NTS |
| --- | --- | --- | --- | --- |
| *staG* | x |  | x |  |
| *tviB* | x | x |  |  |
| *ttr* | x | x | x | x |


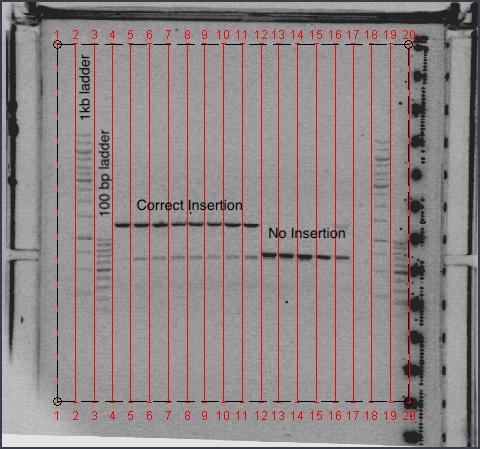


**SI Fig 2: Gel image of the PCR targeting the Tn7 region of the genome.** Lanes containing 1738 bp amplicons indicate bacteria that have the insertion into the genome, while lanes with 678 bp amplicons do not have the insertion.

**SI Checklist 1**

**Ethical considerations, permits and authorship**

*This section is applicable to all research types.*

Provide details as to who granted permissions and/or consent for the study to take place in the Methods section of your manuscript. This should include the names of **all** ethics boards, governmental organizations, community leaders or other bodies that provided approval for the study. If individuals provided approval refer to these people by their role or title but do not list their name(s).

- Sample collection in Seattle, WA was approved by the King County Wastewater Treatment Division research coordinator; this information is reported on page 9 of the submitted manuscript.
- Sample collection in Blantyre was approved by an ethics waiver from the University of Malawi College of Medicine Research Ethics Committee and by the Blantyre City Council; this information is reported on page 10 of the submitted manuscript.
- Sample collection in Vellore was approved by the Vellore City Corporation official and the local elected political representative; this information is reported on page 10 of the submitted manuscript.

If there were any deviations from the study protocol after approval was obtained please provide details of these changes in the Methods section of your manuscript.

- There were no deviations from the study protocol.

Did this study involve local collaborators that are residents of the country where the research was conducted or members of the community studied? If you do not have any authors from said communities, please provide an explanation for this below.

- Yes, this study involved local collaborators that are residents of the countries where research was conducted. They have been included as authors on this manuscript.

**Human subjects research (e.g. health research, medical research, cross-cultural psychology)**

This section is not applicable to the current research project.

**Non-human subjects research using specimens/ animals collected as part of the study, or those housed in archival collectsions. Examples include archaeology, paleontology, botany and zoology.**

Did the permission you obtained from a local authority to perform the study include an agreement on access to outputs and benefit sharing? This may include procedures to enable fair distribution of the benefits and resources arising from the research performed. Please include any details of Prior Informed Consent and Benefit Sharing Agreements obtained. These may be required by field-specific regulations, for example the Convention on Biological Diversity (CBD) and the associated Nagoya Protocol.

- Per our agreement with the King County Wastewater Treatment Division, all manuscripts are shared after publication and data is shared before publication upon request. International study partners share in the benefits of the study by being included as authors on the manuscript.

If the material used in your study was imported, please A) provide the year it was imported and B) indicate whether permits were obtained to import/export the materials used, C) provide details of any permits obtained. If this information is not available, please indicate this.

- Non-infectious DNA was imported to the US. In accordance with Centers for Disease Control and Prevention regulations, these samples are permissible to import without additional permits because the material is not known or suspected to contain an infectious biological agent. The samples were rendered non-infectious through DNA inactivation and extraction using the commercially available Qiagen QIAamp PowerFecal Pro DNA kit. This extraction process uses physical and chemical mechanisms to disrupt the cells, rendering them non-infectious.

If you used archival specimens, please state how the material used in your study was acquired by the institute it is held in and provide details of any permits obtained for the original excavations/ sample collection. If this information is not available, please indicate this.

- No archival specimens were used in this study.

How was the potential cultural significance of the materials collected in your study to local communities considered in your research design? Were Indigenous peoples and/or local researchers and institutions involved with archaeological excavations / collection of specimens? If so, please provide a description of their involvement.

- Local collaborators were involved in the planning and execution process of this study. Given the *S.* Typhi burden of disease in these communities, there was significant local interest in studying wastewater for disease surveillance. In Vellore, our local collaborators worked alongside University of Washington personnel for collection and processing and were additionally trained on the methods.
- In Blantyre, local collaborating institutions were the Malawi Liverpool Wellcome (MLW) Research Programme, an affiliate of the Kamuzu University of Health Sciences.MLW seeks input into study design and permission to work in the community both through the Blantyre City Council (BCC) and through Community Advisory Groups (CAGs). Samples were collected by Malawian Field Researchers and processed in an environmental microbiology laboratory by Malawian laboratory scientists. Results were shared with communities via CAGs, the BCC, and the Blantyre District health office.

If your manuscript includes photographs of human remains please indicate whether authors obtained permission from descendants or affiliated cultural communities to do so.

- Our manuscript does not contain photographs of human remains.

1. McKenzie GJ, Craig NL. Fast, easy and efficient: site-specific insertion of transgenes into Enterobacterial chromosomes using Tn7 without need for selection of the insertion event. BMC Microbiology. 2006;6(1):39.

2. Nga TVT, Karkey A, Dongol S, Thuy HN, Dunstan S, Holt K, et al. The sensitivity of real-time PCR amplification targeting invasive Salmonellaserovars in biological specimens. BMC Infectious Diseases. 2010;10(1):125.

3. Zhou N, Ong A, Fagnant-Sperati C, Harrison J, Kossik A, Beck N, et al. Evaluation of Sampling and Concentration Methods for Salmonella enterica Serovar Typhi Detection from Wastewater. The American Journal of Tropical Medicine and Hygiene. 2023;108(3):482-91.

4. Falman JC, Fagnant-Sperati CS, Kossik AL, Boyle DS, Meschke JS. Evaluation of Secondary Concentration Methods for Poliovirus Detection in Wastewater. Food Environ Virol. 2019;11(1):20-31.
