# Supplementary figures and images for: Development, confirmation, and application of a seeded Escherichia coli process control organism to validate Salmonella enterica serovar Typhi environmental surveillance methods
Source: PLoS One. 2024 May 7;19(5):e0301624. doi: 10.1371/journal.pone.0301624 (PMC11075847; doi:10.1371/journal.pone.0301624)

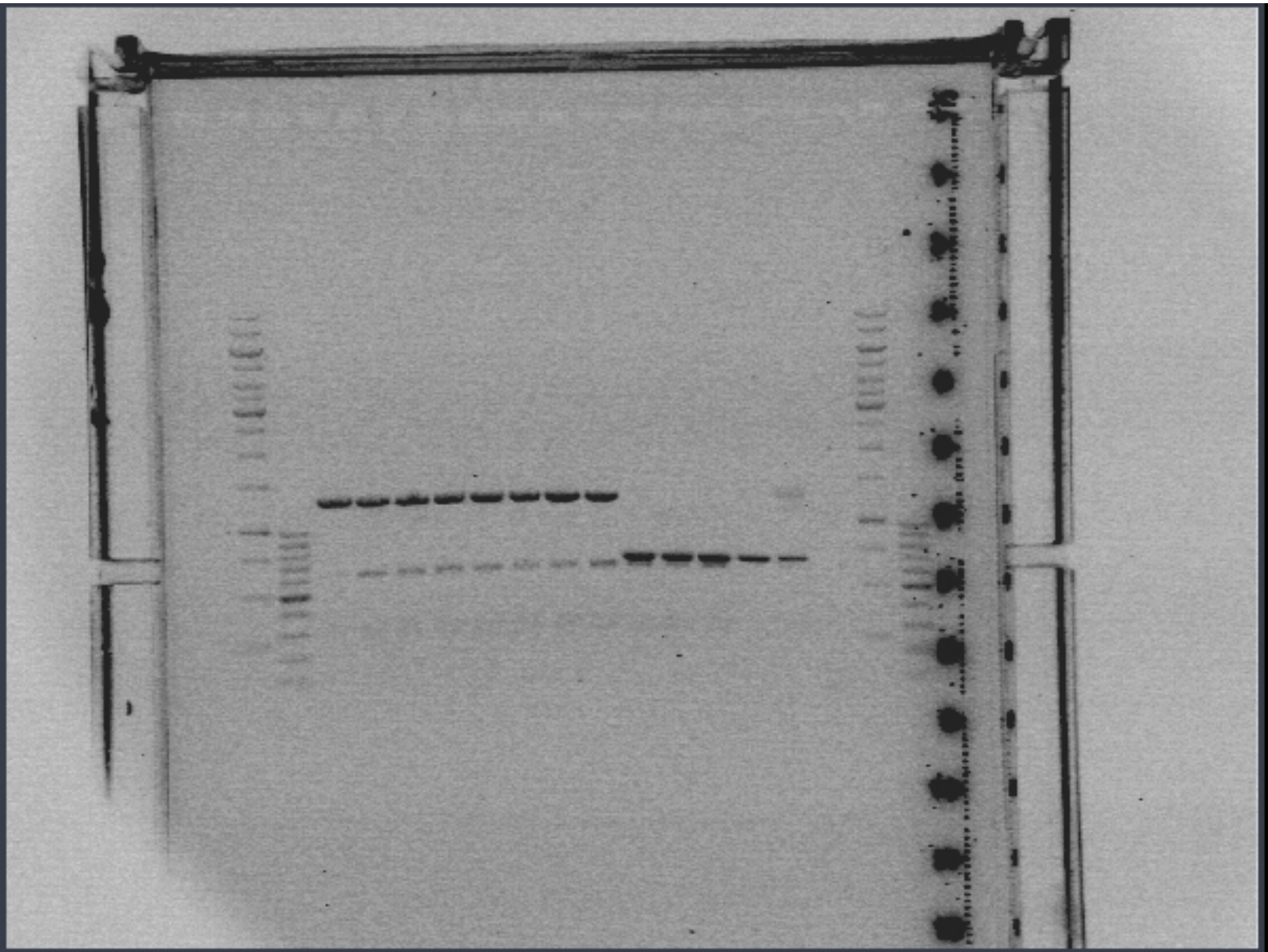

Raw SI Fig. 2

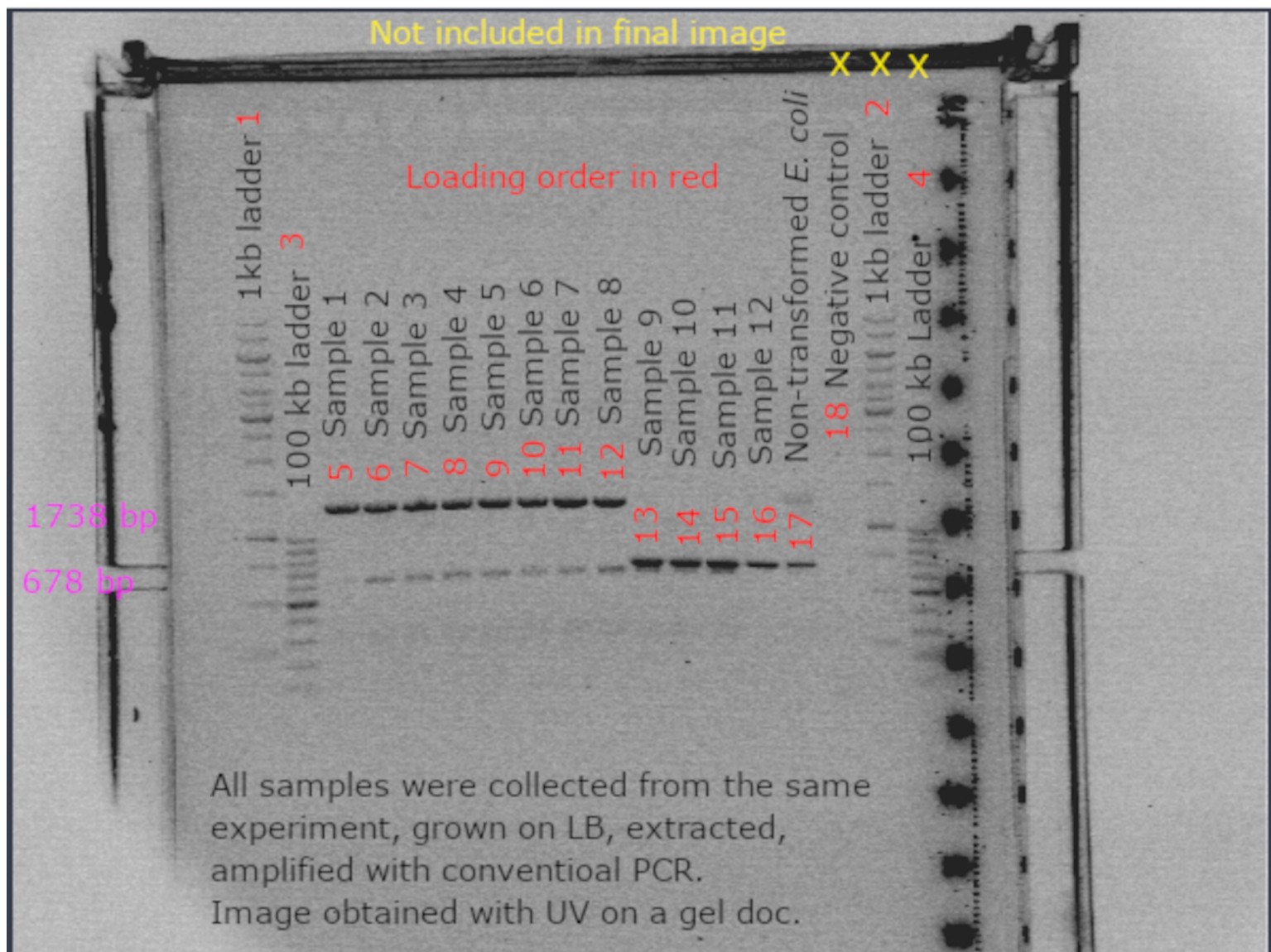

## Annotated SI Fig. 2

Supplement: S1 Raw images — (PDF) [file pone.0301624.s003.pdf]
